# Genetic variants in the plasminogen activator inhibitor‐1 gene are associated with an increased risk of radiation pneumonitis in lung cancer patients

**DOI:** 10.1002/cam4.1011

**Published:** 2017-02-17

**Authors:** Bo Liu, Yang Tang, Minxiao Yi, Qingxu Liu, Huihua Xiong, Guangyuan Hu, Xianglin Yuan

**Affiliations:** ^1^ Department of OncologyTongji HospitalHuazhong University of Science and TechnologyWuhanHubei ProvinceChina

**Keywords:** Lung cancer, PAI‐1, Radiation pneumonitis, Radiotherapy, Single‐nucleotide polymorphism

## Abstract

Plasminogen activator inhibitor‐1 (PAI‐1) plays a crucial role in the process of lung injury, although its association with radiation pneumonitis (RP) is unclear. We hypothesized that genetic variants in *PAI‐1* may influence the risk of RP. In this study, 169 lung cancer patients were genotyped for six single‐nucleotide polymorphisms in *PAI‐1* using the Sequenom MassARRAY system. The risk of RP was evaluated by Cox proportional hazards analyses. The cumulative RP probabilities by genotype were assessed using Kaplan–Meier analyses. Univariate and multivariate analyses revealed that *PAI‐1*:rs7242 GT/GG was correlated with an increased occurrence of grade ≥3 RP (crude hazard ratio = 3.331; 95% confidence interval, 1.168–9.497; *P *=* *0.024). Our results indicated that *PAI‐1*:rs7242 in the 3′‐untranslated region of *PAI‐1* can be a predictor of grade ≥3 RP before radiotherapy.

## Introduction

Radiotherapy is a common modality for treating lung cancer 1. Radiation pneumonitis (RP) is a major side effect associated with radiotherapy which limits the therapeutic ratios of tumor treatment and reduces the living quality in patients who are irradiated for lung cancer. Approximately 16–30% of lung cancer patients experience severe RP after thoracic irradiation 2. Therefore, the exploration and application of RP biomarkers may help maximize efficacy and minimize adverse effects of radiotherapy. Previous studies have investigated and identified multiple therapeutic and patient‐related factors that are associated with the incidence of RP including chemotherapy, smoking status, chronic lung disease, dosimetric parameters, and transforming growth factor (TGF)*β* plasma concentrations 3, 4, 5, 6, 7, 8. However, only a small proportion of patients exposed to similar doses and volumes of irradiation develop RP, indicating that genetic factors perform a crucial role in the RP process. Our previous studies found that single‐nucleotide polymorphisms (SNPs) in the TGF*β* pathway were associated with RP risk 9, 10. Here, we expand upon our previous work by analyzing SNPs in plasminogen activator inhibitor‐1 (*PAI‐1*), an important target gene of TGF*β* with RP risk 11.

PAI‐1 is the main inhibitor of the plasmin system that blocks fibrinolysis and degradation of the extracellular matrix (ECM) 12. PAI‐1 has been implicated in the progression of inflammatory and fibrotic lung diseases. For example, high levels of PAI‐1 in lung edema fluid may affect mortality in acute lung injury 13. In mouse models, inhibiting PAI‐1 expression by small interfering RNA or knockout of *PAI‐1* attenuates bleomycin‐induced lung fibrosis in comparison with wild‐type mice 14, 15. Additionally, a recent study indicated that a truncated PAI‐1 protein (rPAI‐_123_) protects against radiation‐induced lung injury in a murine model 16. Overall, these results implied that PAI‐1 could be involved in the RP process.

Human *PAI‐1* is located on chromosome 7q21.3–q22 and consists of eight introns and nine exons. SNPs in *PAI‐1* may affect the transcriptional activation and plasma concentrations of PAI‐1 17. Previous studies demonstrated that *PAI‐1* polymorphisms were associated with keloids, susceptibility to idiopathic interstitial pneumonia, myocardial infarction, and lung cancer prognosis 18, 19, 20, 21. However, no studies have examined how *PAI‐1* polymorphisms influence the risk of RP. Here, we investigated the association of SNPs in *PAI‐1* with RP risk in lung cancer patients treated with radiotherapy.

## Materials and Methods

### Study populations

This prospective study (NCT02490319) included 169 lung cancer patients. All patients received radiotherapy between September 2008 and June 2014 at Tongji Hospital (Wuhan, China). The enrolled patients had an expected survival > 6 months, Karnofsky Performance Status > 60, and received a radiation dose more than 45 Gy. Exclusion criteria included respiratory infection or lung fibrosis, pulmonary emboli, cardiac disease, drug toxicity, and previous thoracic irradiation. The Tongji Hospital Review Board approved our study. All patients enrolled in the study signed written informed consents for DNA and clinical information.

All enrolled patients underwent radiotherapy with a 6‐MV linear accelerator (Elekta, Stockholm, Sweden). The total radiation dose was reached by administering 1.5–2 Gy per treatment. Dose–volume histogram data were shown in Table S1. Seventy‐nine patients received intensity‐modulated radiation therapy. One hundred sixty patients received induction chemotherapy followed by radiation or concurrent chemotherapy and radiation, with 32.9% receiving a gemcitabine/cisplatin regimen, 19% a CPT‐11/cisplatin regimen, 19.6% a docetaxel/cisplatin regimen, and 15.8% an etoposide/cisplatin regimen. We used a three‐dimensional planning system (Pinnacle software, version 9.2; Philips Healthcare, Cleveland, OH) to delineate critical normal organs and target volumes.

Details of the follow‐up schedule and the RP scoring criteria have been described previously 10. Briefly, RP was diagnosed by two radiation oncologists after reviewing chest X‐ray or computed tomography scans, pulmonary function tests, and clinical information, including symptoms, at each follow‐up visit. The patients were followed during and 1 month after therapy, then every 3 months. RP was scored according to the Common Terminology Criteria for Adverse Events 4.0. Symptomatic RP interfering with daily activities, or a requirement for oxygen, were defined as grade 3.

### Genotyping methods

Genomic DNA from all patients was extracted from peripheral blood via a blood DNA Kit (K1820‐01; Invitrogen, Carlsbad, CA). Based on the public HapMap SNP database and HaploView 4.2 software, we searched for SNPs in *PAI‐1* that had minor allele frequencies greater than 10%, positioned within the 15‐kb region or in its upstream or downstream regulatory regions. We found that all eligible SNPs could be captured with r^2^ > 0.8 by five tagged SNPs: rs2227631, rs2227667, rs2227672, rs2227692, and rs7242. Together with the well‐studied functional SNP rs1799768 (or 4G5G) 20, six SNPs in *PAI‐1* were selected (Table 1). The SNPs were genotyped by the Sequenom MassARRAY system (Agena Bioscience, San Diego, CA) as described previously 10.

**Table 1:** Characteristics of six SNPs selected for analysis

SNP ID	Chromosome	Position	Allele	Function class	
rs2227631	7	101126257	G>A	promoter	tagSNP
rs1799768	7	101126425	–>G	promoter	
rs2227667	7	101131468	A>G	intron 3	tagSNP
rs2227672	7	101132405	G>T	Intron 4	tagSNP
rs2227692	7	101135963	C>T	Intron 7	tagSNP
rs7242	7	101138164	G>T	3′‐UTR	tagSNP

SNP, single‐nucleotide polymorphisms; UTR, untranslated region.

### Statistical analyses

The time for developing grade ≥3 RP was the endpoint used for this analysis. Data from patients were censored if they did not develop grade ≥3 RP within 1 year. SPSS version 19.0 (IBM, Chicago, IL) was used for statistical analyses. The Cox proportional hazards model was applied to estimate hazard ratios with 95% confidence intervals of different genotypes. Multivariate Cox regression analysis was used to adjust other covariates. Kaplan–Meier analyses were used to evaluate influences of the genotypes on RP between groups by log‐rank tests. *P *<* *0.05 was considered statistically significant in all tests.

## Results

### Patient characteristics and association with RP

Table 2 lists characteristics of the 169 (125 male and 44 female) lung cancer patients (114 non‐small‐cell lung carcinoma and 55 small‐cell lung carcinoma). The median age of patients was 57 years (28–78 years). One hundred six (62.0%) of the patients were smokers. Among the 169 patients, 145 (85.8%) had stage III–IV disease, 160 (94.7%) were treated with chemotherapy, and 86 (50.9%) underwent surgery before radiotherapy.

**Table 2:** Patient characteristics (*n* = 169)

Characteristics	No. of Patients	%
Sex		
Male	125	74.0
Female	44	26.0
Age, years		
Median	57	
Range	28–78	
Histology		
SCLC	55	32.5
NSCLC	114	67.5
Stage		
I‐ II	24	14.2
IIIA	81	47.9
IIIB	46	27.2
IV	18	10.7
KPS		
80‐100	123	72.6
<80	46	27.4
Smoking		
Smoker	106	62.0
Nonsmoker	63	38.0
Chemotherapy		
Yes	160	94.7
No	9	5.3
Type of chemotherapy		
Sequential	119	74.4
Concurrent	41	25.6
CRT		
Yes	44	26.0
No	125	74.0
Surgery		
Yes	86	50.9
No	83	49.1
IMRT		
Yes	79	46.7
No	90	53.3
Radiation dose (cGy)		
Median	5600	
Range	4500–6600	
MLD (cGy)		
Median	1368	
Range	178–2017	
V_20_		
Median	24.82	
Range	0–42.00	
COPD		
Yes	19	11.2
No	150	88.8

KPS, Karnofsky performance status; CRT, concurrent chemoradiation; IMRT, intensity‐modulated radiation therapy; MLD, mean lung dose; V_20_, volume of normal lung receiving 20 Gy or more radiation; COPD, chronic obstructive pulmonary disease.

Smoker is the person who has or had smoked for more than 6 months, including former smoker and current smoker.

The median follow‐up time in this study was 22 months (6–52 months). After treatment with radiotherapy, 32 patients (18.9%) had grade ≥3 RP (grades 3, 4, and 5 were found in 29, 1, and 2 patients, respectively). We evaluated the association between clinicopathologic characteristics and grade ≥3 RP risk. According to multivariate analysis, V_5_ ≥48%, V_10_ ≥ 38%, V_20_ ≥ 24% and a mean lung dose (MLD) ≥15 Gy were associated with increased grade ≥ 3 RP risk (*P *=* *0.009, *P *=* *0.019, *P *=* *0.034, and *P *=* *0.014, respectively). None of the other clinicopathologic characteristics were associated with a risk of RP in this study (Table S1 and Table 3).

**Table 3:** Association between patient characteristics and grade ≥3 RP

Parameter	Univariate analysis			Multivariate analysis		
	HR	95%CI	*P*	HR	95%CI	*P*
Sex						
Female	1			1		
Male	1.604	0.660–3.897	0.297	1.425	0.450–4.513	0.547
Age, years						
<57	1			1		
≥57	1.838	0.886–3.813	0.102	2.098	0.967–4.554	0.061
Histology						
SCLC	1			1		
NSCLC	1.071	0.507–2.261	0.858	1.788	0.736–4.339	0.199
Stage						
I–II	1			1		
III–IV	0.877	0.308–2.501	0.806	0.758	0.250–2.297	0.624
KPS						
80–100	1			1		
<80	1.066	0.493–2.305	0.870	0.867	0.381–1.973	0.734
Smoking						
Smoker	1			1		
Nonsmoker	0.670	0.317–1.414	0.293	0.883	0.326–2.932	0.807
Surgery						
Yes	1			1		
No	1.383	0.688–2.781	0.363	1.097	0.520–2.315	0.807
Chemotherapy						
Sequential	1	1		1		
Concurrent	1.503	0.703–3.210	0.293	1.562	0.710–3.437	0.267
No	1.457	0.341–6.236	0.611	1.599	0.358–7.144	0.539
CRT						
Yes	1			1		
No	0.648	0.312–1.344	0.244	0.508	0.029–8.790	0.642
IMRT						
Yes	1			1		
No	1.029	0.514–2.059	0.937	1.115	0.523–2.376	0.778
Radiation dose, cGy						
<5600	1			1		
≥5600	1.294	0.639–2.621	0.473	1.108	0.526–2.334	0.788
MLD, cGy						
<1500	1			1		
≥1500	2.353	1.175–4.714	**0.016**	2.540	1.207–5.347	**0.014**
V20						
<24%	1			1		
≥24%	2.334	1.049–5.197	**0.038**	1.599	1.070–5.860	**0.034**
COPD						
Yes	1			1		
No	0.639	0.246–1.661	0.358	0.602	0.224–1.617	0.314

Multivariate analyses were adjusted for sex, age, smoking, surgery, chemotherapy, and V_20_.

HR, hazard ratio; KPS, Karnofsky performance status; RT, radiotherapy; CRT, concurrent chemoradiation; IMRT, intensity‐modulated radiation therapy; MLD, mean lung dose; V_20_, volume of normal lung receiving 20 Gy or more radiation; COPD, chronic obstructive pulmonary disease.

MLD and V_20_ were not used together in multivariate analyses.

*P* < 0.05 are presented in bold.

### RP and *PAI‐1* polymorphisms

**Figure 1:**
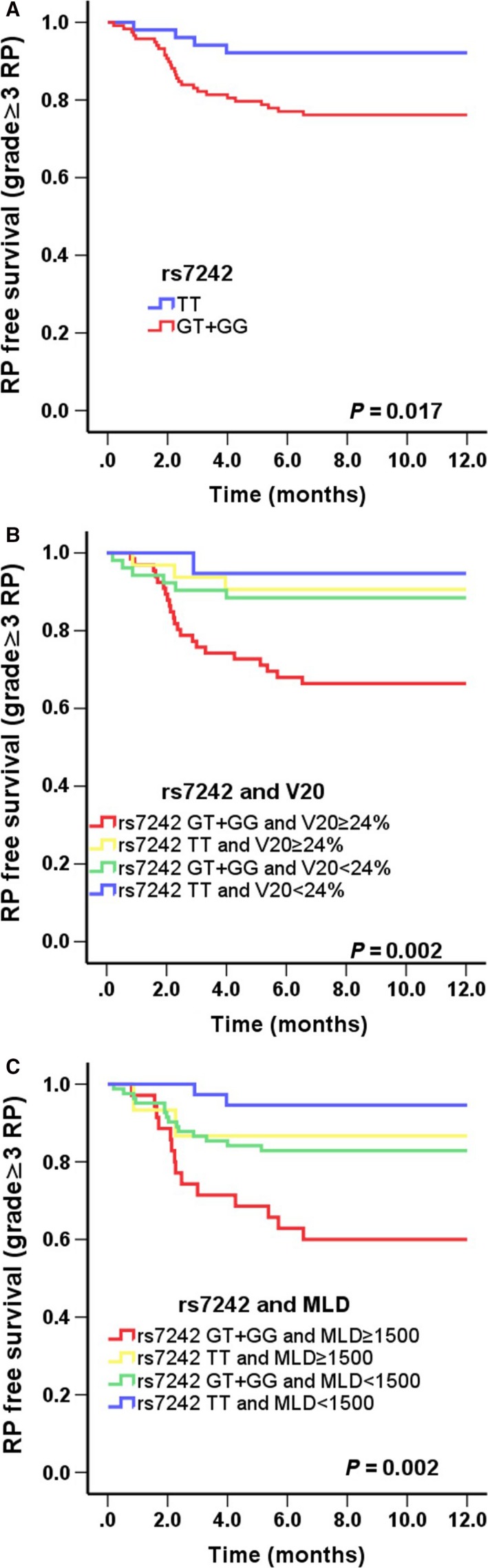
Kaplan–Meier estimates RP‐free survival (RP ≥ grade 3) as a function of time from the start of radiation therapy by genotypes. (A) rs7242; (B) rs7242 and V20; (C) rs7242 and MLD. The rs7242 GT/GG genotypes was associated with a significantly higher risk of RP as compared with TT genotype (*P *=* *0.017). Patients with GT/GG genotype of rs7242 and V20 ≥ 24% or MLD ≥ 15 Gy had the highest grade ≥3 RP risk compared with other groups.

The associations between genetic polymorphisms and the risk of grade ≥3 are shown in Table 4 using the Cox proportional hazards model. A significant association was found between rs7242 and the risk of grade ≥3 RP. Compared with the rs7242 TT genotype, the GT/GG genotypes had increased hazards of grade ≥3 RP (*P = *0.024). We found a similar result after multivariate analyses with adjustment for potential confounding factors of RP. The RP‐free survival for grade ≥3 RP, according to rs7242 is plotted in Figure 1A. Development of grade ≥3 RP was prolonged in the rs7242 GG/GT genotypes, while no associations with grade ≥3 RP were found for the other SNPs.

**Table 4:** Association between *PAI‐1* genotypes and grade ≥3 RP

Polymorphism and Genotype	No. of event	No. of total	Univariate analysis			Multivariate analysis		
			HR	95% CL	*P*	HR	95% CL	*P*
*PAI‐1:rs2227631*								
GG	10	64	1			1		
AG	15	81	1.208	0.543–2.688	0.644	1.564	0.695–3.519	0.279
AA	7	21	2.389	0.909–6.28	0.077	2.636	0.982–7.076	0.054
AA+AG	22	102	1.433	0.679–3.027	0.345	1.794	0.840–3.829	0.131
*PAI‐1:rs1799768*								
4G/4G	12	71	1					
4G/5G	16	76	1.247	0.590–2.636	0.563	1.403	0.651–3.022	0.387
5G/5G	3	21	0.848	0.239–3.004	0.798	1.111	0.278–3.875	0.956
5G/5G+4G/5G	19	97	1.161	0.563–2.391	0.686	1.339	0.633–2.834	0.445
*PAI‐1:rs2227667*								
AA	13	54	1			1		
AG	18	85	0.867	0.425–1.770	0.695	0.816	0.390–1.710	0.590
GG	1	29	0.130	0.017–0.992	0.049	0.115	0.014–0.924	0.042
GG+AG	19	114	0.668	0.330–1.352	0.262	0.658	0.313–1.385	0.270
*PAI‐1:rs2227672*								
GG	27	135	1			1		
GT	5	34	0.742	0.286–1.927	0.540	0.677	0.249–1.842	0.445
*PAI‐1:rs2227692*								
CC	18	76	1			1		
CT	14	74	0.777	0.387–1.563	0.479	0.829	0.396–1.735	0.619
TT	0	17	NC	NC	0.971	NC	NC	0.971
CT+TT	14	91	0.620	0.308–1.246	0.179	0.673	0.316–1.433	0.304
*PAI‐1:rs7242*								
TT	4	51	1			1		
GT	15	76	2.710	0.899–8.165	0.077	3.558	1.127–11.23	**0.030**
GG	13	42	4.532	1.477–13.90	**0.008**	5.200	1.623–16.66	**0.006**
GG+GT	28	118	3.331	1.168–9.497	**0.024**	4.188	1.404–12.50	**0.010**

Multiple analyses in this table were adjusted for sex, age, smoking, surgery, chemotherapy, and V_20_.

PAI‐1, Plasminogen activator inhibitor‐1; HR, hazard ratio.

*P* < 0.05 are presented in bold.

*NC* not calculated.

### *PAI‐1:rs7242* and dosimetric factors

The cumulative probability of grade ≥3 RP on the basis of genotype and V_20_ as a function of time is shown in Figure 1B. The incidence of RP in patients receiving V_20_ ≥ 24% and GT/GG genotypes in rs7242 were higher than patients who received V_20_ ≥ 24% with the TT genotype in rs7242 (*P *=* *0.013). We also analyzed the cumulative RP incidence on the basis of MLD and genotypes as a function of time (Fig. 1C). Patients with a MLD ≥ 15 Gy and GT/GG genotypes in rs7242 displayed a higher RP hazard than patients with the TT genotype and a MLD ≥ 15 Gy (*P *=* *0.010). However, we did not observe this difference in patients who received V_20_ < 24% or a MLD < 15.0 Gy. These results suggest the independent role of rs7242 genotypes in grade ≥3 RP.

## Discussion

This study examined whether genetic polymorphisms in *PAI‐1* gene might be associated with an increased risk of RP in lung cancer patients receiving radiotherapy. We believe this is the first finding of an association between the presence of rs7242 in the 3′‐ untranslated region (UTR) of *PAI‐1* and the risk of grade ≥3 RP. We found that patients with the rs7242 GG or GT genotypes exhibited an increased risk of RP following radiotherapy. Our results also indicated that the association between rs7242 and grade ≥ 3 RP risk was independent of V_20_ and MLD. Moreover, a group of patients (GT/GG genotypes in rs7242 and V_20_ ≥ 24% or MLD ≥ 15 Gy) were found with the highest occurrence of grade ≥3 RP.

RP is a common complication following radiotherapy and is characterized by diffuse alveolar damage and subsequent fibrosis with excessive ECM deposition in the lung 22. PAI‐1 is the main inhibitor of the plasmin system and has a crucial role in ECM accumulation by inhibiting fibrinolysis 12. Although little is known about the association between *PAI‐1* polymorphisms and RP risk, several facts indicate that this association is biologically plausible. First, genetic variants in *PAI‐1* influence the plasma levels of PAI‐1 and are associated with other inflammatory or fibrotic diseases including keloids, myocardial infarction, and idiopathic interstitial pneumonia 18, 19, 20. Second, PAI‐1 is implicated in the development of other radiation injury diseases. For example, there is a high level of PAI‐1 in radiation‐induced nephrosclerosis and the process of radiation enteritis 23, 24. *PAI‐1* knockout mice have better survival and intestinal function compared with wild‐type mice in radiation‐induced intestinal injury 25. Finally, *PAI‐1* is closely regulated by TGF‐*β*1, the cytokine that has a critical role in the RP process 22, 26. TGF‐*β*1 can regulate PAI‐1 expression via SMAD‐dependent and ‐independent pathways in numerous fibrotic diseases 11, 27, 28, 29, 30, 31. Moreover, TGF‐*β*1 increases PAI‐1 plasma levels and promotes the epithelial‐mesenchymal transition (EMT), while PAI‐1 small interfering RNA prevents the TGF‐*β*1‐induced EMT in mouse lung epithelial cells 12.

In this study, rs7242 was associated significantly with grade ≥3 RP. The rs7242 polymorphism is located in the 3′‐UTR of *PAI‐1* and is characterized by the substitution of a guanine with thymine. Studies have examined the relationships between this polymorphism and the risk of myocardial infarction, diffuse‐type gastric cancer susceptibility, and primary ovarian insufficiency 19, 32, 33. In addition, previous studies found that haplotypes of this polymorphism may affect the plasma level of PAI‐1 32, 34. Other research reported that the rs7242 polymorphism may affect blood insulin concentrations 19. Because insulin levels play particular roles in lung diseases 35, rs7242 may also modulate the risk of RP by influencing insulin levels in cancer patients.

In summary, these facts suggest that the influence of rs7242 on RP is biologically plausible. However, in this study, we did not observe that other *PAI‐1* polymorphisms affected the risk of RP. This included rs1799768 that can influence PAI‐1 plasma levels and confer an increased risk of several inflammatory or fibrotic diseases such as myocardial infarction, asthma, nephropathy, and idiopathic interstitial pneumonia 20, 36, 37, 38. This finding may have been due to the different nature of the diseases and the small size of the study population.

Our study suggested that the rs7242 polymorphism can be used as a predictor of RP. In combination with our previous findings concerning RP susceptibility and SNPs in *TGFβ1, ITGB6, PI3CA, AKT2*, and *MMP1* 9, 10, 39, 40, we can establish a more accurate model using these variants, enabling the prediction of RP by genotyping patients prior to radiotherapy. This would enable patients lacking RP susceptibility genotypes to receive appropriately elevated radiation doses to enhance tumor‐related therapies.

In spite of these positive findings, some limitations of our study should be addressed. First, the population of this study was relatively small and thus the results need to be confirmed by further validation. Moreover, we were unable to explore the exact mechanism by which *PAI‐1* polymorphisms led to RP in lung cancer patients. Finally, as the power in this exploratory study was limited, the *P* values in this study were not adjusted using Bonferroni corrections. Therefore, our findings are considered preliminary.

In conclusion, this study identified that rs7242 GT/GG genotypes located in the 3′‐UTR of *PAI‐1* were significantly associated with an increased risk of RP in lung cancer patients treated with radiotherapy. Our findings suggested that this polymorphism could be used to predict RP in lung cancer patients prior to initiating radiotherapy. However, further studies are essential to confirm our findings.

## Conflict of Interest

The authors have no conflict of interests.

## Supporting information


**Table S1**. Association between Dose–volume histogram data and grade ≥ 3 RP.
**Table S2**. Association between *PAI‐1* genotypes and grade ≥ 2 RP.
