# Supplementary material for: Genetic variants in the plasminogen activator inhibitor‐1 gene are associated with an increased risk of radiation pneumonitis in lung cancer patients
Source: Cancer Med. 2017 Feb 17;6(3):681–8. doi: 10.1002/cam4.1011 (PMC5345627; doi:10.1002/cam4.1011)
Supplement: Supplementary file 1 — Table S1. Association between Dose–volume histogram data and grade ≥ 3 RP. Table S2. Association between PAI‐1 genotypes and grade ≥ 2 RP. [file CAM4-6-681-s001.docx]

| **Supplementary Table1.** **Association between** **Dose-volume Histogram data and grade ≥ 3 RP** | | | | | | | | | |
| --- | --- | --- | --- | --- | --- | --- | --- | --- | --- |
| **Parameter** |  | | **Univariate Analysis** | | | | **Multivariate Analysis** | | |
|  |  | **HR** | | **95%CI** | **P** | **HR** | | **95%CI** | **P** |
| **V_5_****(n=167)**  Median  Range | <48%  ≥48%  48.00  5-75.00 | 1  2.827 | | 1.264-6.322 | **0.011** | 1  2.982 | | 1.316-6.757 | **0.009** |
| **V_10_(n=136)**  Median  Range | <38%  ≥38%  37.75  1-55.00 | 1  2.206 | | 0.899-5.412 | 0.084 | 1  3.221 | | 1.211-8.570 | **0.019** |
| **V_20_(n=169)**  Median  Range | <24%  ≥24%  24.39  0-42.00 | 1  2.334 | | 1.049-5.197 | **0.038** | 1  1.599 | | 1.070-5.860 | **0.034** |
| **V_30_(n=165)**  Median  Range | <18%  ≥18%  17.70  2-35.00 | 1  1.235 | | 0.609-2.506 | 0.558 | 1  0.798 | | 0.244-2.607 | 0.567 |

NOTE: Multiple analyses in this table were adjusted for sex, age, smoking, surgery, chemotherapy.

| **Supplementary Table2. Association between *PAI-1* genotypes and grade ≥ 2 RP** | | | | | | | | | |
| --- | --- | --- | --- | --- | --- | --- | --- | --- | --- |
| Polymorphism and Genotype | | No.of event | No.of total | Univariate analysis | | | Multivariate analysis | | |
|  |  |  |  | HR | 95% CL | P | HR | 95% CL | P |
| *PAI-1:rs2227631* | |  |  |  |  |  |  |  |  |
| GG  AG  AA  AA+AG | | 37  47  14  61 | 64  81  21  102 | 1  1.008  1.629  1.104 | 0.655-1.550  0.880-3.015  0.734-1.661 | 0.972  0.120  0.634 | 1  1.242  1.870  1.355 | 0.789-1.954  0.977-3.580  0.884-2.079 | 0.349  0.059  0.163 |
| *PAI-1:rs1799768* | |  |  |  |  |  |  |  |  |
| 4G/4G | | 40 | 71 | 1 |  |  |  |  |  |
| 4G/5G | | 46 | 76 | 1.113 | 0.728-1.700 | 0.621 | 1.273 | 0.662-1.689 | 0.281 |
| 5G/5G | | 12 | 21 | 1.165 | 0.611-2.222 | 0.642 | 1.320 | 0.666-2.612 | 0.426 |
| 5G/5G+4G/5G | | 58 | 97 | 1.123 | 0.751-1.680 | 0.572 | 1.281 | 0.842-1.919 | 0.247 |
| *PAI-1:rs2227667* | |  |  |  |  |  |  |  |  |
|  | GG | 13 | 29 | 1 |  |  | 1 |  |  |
|  | AG | 55 | 85 | 1.487 | 0.778-2.841 | 0.071 | 1.617 | 0.852-3.070 | 0.141 |
|  | AA | 31 | 54 | 1.746 | 0.954-3.198 | 0.230 | 1.408 | 0.696-2.850 | 0.341 |
|  | AA+AG | 86 | 139 | 1.634 | 0.916-2.944 | 0.096 | 1.548 | 0.828-2.893 | 0.171 |
| *PAI-1:rs2227672* | |  |  |  |  |  |  |  |  |
|  | GG | 83 | 135 | 1 |  |  | 1 |  |  |
|  | GT | 16 | 34 | 0.697 | 0.408-1.191 | 0.187 | 0.559 | 0.315-0.991 | **0.046** |
| *PAI-1:rs2227692* | |  |  |  |  |  |  |  |  |
|  | CC | 42 | 76 | 1 |  |  | 1 |  |  |
|  | CT | 50 | 74 | 1.356 | 0.898-2.046 | 0.147 | 1.638 | 1.044-2.570 | 0.032 |
|  | TT | 7 | 17 | 0.616 | 0.277-1.370 | 0.235 | 0.738 | 0.308-1.767 | 0.459 |
|  | CT+TT | 57 | 91 | 1.179 | 0.791-1.758 | 0.418 | 1.458 | 0.937-2.270 | 0.095 |
| *PAI-1:rs7242* | |  |  |  |  |  |  |  |  |
|  | TT | 25 | 51 | 1 |  |  | 1 |  |  |
|  | GT  GG  GG+GT | 46  28  74 | 76  42  118 | 1.399  1.697  1.499 | 0.860-2.278  0.989-2.911  0.952-2.359 | 0.176  0.055  0.080 | 1.571  1.907  1.685 | 0.926-2.668  1.066-3.410  1.027-2.765 | 0.094  **0.030**  **0.039** |

NOTE: Multiple analyses in this table were adjusted for sex, age, smoking, surgery, chemotherapy and V_20_.

Abbreviations: PAI-1, Plasminogen activator inhibitor-1; HR, hazard ratio.
